# Sn^2+^ Doping: A Strategy for Tuning of Fe_3_O_4_ Nanoparticles Magnetization Dipping Temperature/Amplitude, Irreversibility, and Curie Point

**DOI:** 10.1186/s11671-020-03423-9

**Published:** 2020-10-01

**Authors:** Umaima S. H. Al-Kindi, Salim H. Al-Harthi, Hisham M. Widatallah, Mohamed E. Elzain, Myo T. Z. Myint, Htet H. Kyaw

**Affiliations:** 1 Department of Physics, College of Science, Sultan Qaboos University, P.O. Box 36, Al-Khoudh, Muscat, 123 Sultanate of Oman; 2 Nanotechnology Research Center, Sultan Qaboos University, P.O. Box 33, Al-Khoudh, Muscat, 123 Sultanate of Oman

**Keywords:** Curie, Dipping, Irreversible, Maghemite, Magnetite, Modified Bloch law

## Abstract

Doped magnetite (Sn_*x*_Fe_3-2/3*x*_O_4_) nanoparticles (NPs) (12–50 nm) with different amount of Sn^2+^ ions (*x*) were synthesized using co-precipitation method. Sn^2+^ doping reduces the anticipated oxidation of Fe_3_O_4_ NPs to maghemite (γ-Fe_2_O_3_), making them attractive in several magnetic applications. Detailed characterizations during heating–cooling cycles revealed the possibility of tuning the unusual observed magnetization dipping temperature/amplitude, irreversibility, and Curie point of these NPs. We attribute this dip to the chemical reduction of γ-Fe_2_O_3_ at the NPs surfaces. Along with an increase in the dipping temperature, we found that doping with Sn^2+^ reduces the dipping amplitude, until it approximately disappears when *x* = 0.150. Based on the core-shell structure of these NPs, a phenomenological expression that combines both modified Bloch law (*M* = *M*_0_[1 − *γ*(*T*/*T*_*C*_)]^*β*^) and a modified Curie–Weiss law (*M* = − *α*[1/(*T* − *T*_*C*_)^*δ*^]) is developed in order to explain the observed *M*-*T* behavior at different applied external magnetic fields and for different Sn^2+^ concentrations. By applying high enough magnetic field, the value of the parameters *γ* and *δ* ≈ 1 which are the same in modified Bloch and Curie–Weiss laws. They do not change with the magnetic field and depend only on the material structure and size. The power *β* for high magnetic field was 2.6 which is as expected for this size of nanoparticles with the core dominated magnetization. However, the *β* value fluctuates between 3 and 10 for small magnetic fields indicating an extra magnetic contribution from the shell structure presented by Curie–Weiss term. The parameter (*α*) has a very small value and it turns to negative values for high magnetic fields.

## Introduction

Metal oxide nanoparticles are attractive from both technical and theoretical perspective. Among them, iron oxide nanoparticles are very popular due to their massive applications in the fields of ferrofluids, pigments, information storage disks, and medical applications as magnetically guided drug delivery, cell separation, and cancer diagnoses [1–9]. Magnetite (Fe_3_O_4_) nanoparticles are particularly well suited for medical applications, due to their biological compatibility and the large saturation magnetization (M_s_) of 92 emu/g at 300 K for the bulk [10, 11]. However, the thermal instability of these nanoparticles can be a drawback for these applications since nanoparticles with size of ~ 8–22 nm can be easily oxidized to maghemite (γ-Fe_2_O_3_) even at ambient conditions of temperature and pressure—although the bulk can be accomplished at ~ 220 °C [12]. Maghemite is a ferrimagnetic material like magnetite with the same spinel structure but with lower M_s_ of 78 emu/g at 300 K [10]. By heating up to about 850 K (Curie point), Fe_3_O_4_ can be structurally changed to the antiferromagnetic corundum-like structure hematite with zero M_s_ [13]. These transformations are controlled by particle size, temperature, and pressure. Scarce studies are made for Fe_3_O_4_ particles at high temperature because of thermal instability. Recently more attention has been given to the effects of organic capping—such as an oleate-capped Fe_3_O_4_ nanoparticles—on the magnetization of the nanoparticles (NPs) [14]. It was found that, in heating–cooling cycles, Fe_3_O_4_ NPs exhibited irreversible *M* behavior with two peculiar effects, namely, dips and loops in their *M* (*T*) curves. The dipping and the irreversible magnetization were attributed to the induced reduction of Fe^3+^ to Fe^2+^ and sintering upon the decomposition of the capping ligands respectively. Our intention in this study is to thoroughly understand the cause of these peculiar effects, their nature, stability, effects on magnetization, and surface reduction of Fe^3+^ to Fe^2+^ and their relationship with sintering process of the NPs at elevated temperatures. Motivated by the fact that Fe_3_O_4_ NPs can be easily oxidized to form γ-Fe_2_O_3_ shell (i.e., thin layer thereafter called a shell) on the surface which acts as a capping layer and exploiting the knowledge that doping Fe_3_O_4_ with certain ions like Sn^4+^ and Ti^4+^ shows a decrease in the Fe^3+^ to Fe^2+^ reduction process [15, 16], we therefore explore the possibility of tuning those peculiar effects (i.e., dipping and loops) in temperature dependent magnetization curves by Sn^2+^ doping of Fe_3_O_4_ NPs.

In order to study the effect of Sn^2+^ doping on the stability of magnetite nanoparticles, magnetization dipping, and irreversibility at high temperatures, Sn_*x*_Fe_3-2/3*x*_O_4_ nanoparticles (12–50 nm) with (*x* = 0.000, 0.045, 0.090, and 0.150), were prepared and characterized using several complementary techniques. The magnetization was measured using a vibrating sample magnetometer (VSM) while repeatedly heating the sample up to 900 K (5 K/min) and cooling back to room temperature (300 K). An irreversible dip in magnetization was noticed at a specific temperature and with certain amplitude during the first heating–cooling cycle. Evidences of the change in dipping temperature, and amplitude, irreversibility, divergence in magnetization (i.e., magnetization values are different at specific temperature in heating and cooling cycles) and Curie point with *x* were observed and explained. Contrary to the explanation that the observed irreversibility in heating–cooling regime can only be expected for the ligand-free Fe_3_O_4_ NPs, we show that divergence can be controlled by the external magnetic field applied to the Fe_3_O_4_ NPs during the magnetic measurements and disappears at higher applied field. Furthermore, we show that the *M*-*T* of the pristine and the Sn^2+^-doped Fe_3_O_4_ NPs after the first heating–cooling cycle can be predicted by a new approach that combines both a modified Bloch and Curie–Weiss laws for different Sn^2+^ concentrations and different applied external magnetic fields.

## Methods/Experimental

### Materials

Aqueous ammonia (Mw = 17.03, 30%) and absolute ethanol were purchased from Merck, ferric chloride hexahydrate (Mw = 270.3, ≥ 99%) and ferrous chloride tetrahydrate (Mw = 198.8, ≥ 99%) were procured from Sigma-Aldrich, and stannous chloride (Mw = 189.60, ≥ 98%) was obtained from Fluka. All the chemicals were used without further purification.

### Methods

Nanoparticles of Sn^2+^ doped Fe_3_O_4_ with the nominal composition Sn_*x*_Fe_3-2/3*x*_O_4_ (*x* = 0.000, 0.045, 0.090, and 0.150), where Sn^2+^ substitutes Fe^3+^, were prepared using co-precipitation under reflux at 80 ^°^C for 4 h. Aqueous ammonia was added to stoichiometric solutions of ferric chloride hexahydrate, ferrous chloride tetrahydrate, and stannous chloride at 50 °C until a pH ≈ 10.4 was attained. The precipitates were then removed by filtration, washed with distilled water followed by ethanol, and very carefully dried at room temperature avoiding high temperature that would result in the formation of Sn-doped maghemite as was demonstrated by Berry et al. [16].

The surface of pristine Fe_3_O_4_ nanoparticles was covered with 2 nm layer of gold (99.99% gold target, Scotech) using e-beam evaporation (deposition rate ~ 0.47 Å/s) attached to the Nanosys 550 nanoparticle deposition system from Mantis Deposition Ltd. in order to examine the surface effect.

### Characterizations

A VSM attached to a quantum design physical property measurement system (Dynacool PPMS) was used for magnetic measurements at temperature ranging from 2 to 900 K with magnetic fields up to 9 (Tesla). Curie point was taken by the extrapolation of M curve to the *x*-axis during the first heating regime following the procedure used in reference [17]. The morphology of the samples was characterized using JOEL digital high-resolution (JEN-2100F) transmission electron microscope (HRTEM) and (X’Pert PRO) diffractometer for X-ray powder diffraction (XRD) patterns using a standard Cu-Kα radiation. The MAUD software was used to perform simple XRD Rietveld refinements [18]. Elemental mapping (EDX) was carried out using field emission scanning electron microscope (JOEL, JSM 7600F). X-ray photoemission spectra (XPS) were acquired using Omicron Nanotechnology multiprobe photoelectron instrument equipped with a hemispherical electron analyzer where Al Kα radiation (1486.6 eV) was used at 10^−9^ mbar. Intrinsic carbon peak at 284.6 eV was employed for calibration. The Casa XPS software was used for XPS data analysis [19]. Fourier transform infrared (FTIR) spectrum was obtained from PerkinElmer (SpectraOne) using transmission mode with KBr pellets in the range of 400–4000 cm^−1^.

## Results and Discussion

### The Main Features of *M*-*T* Curves During the First Heating Cycle

**Fig. 1:**
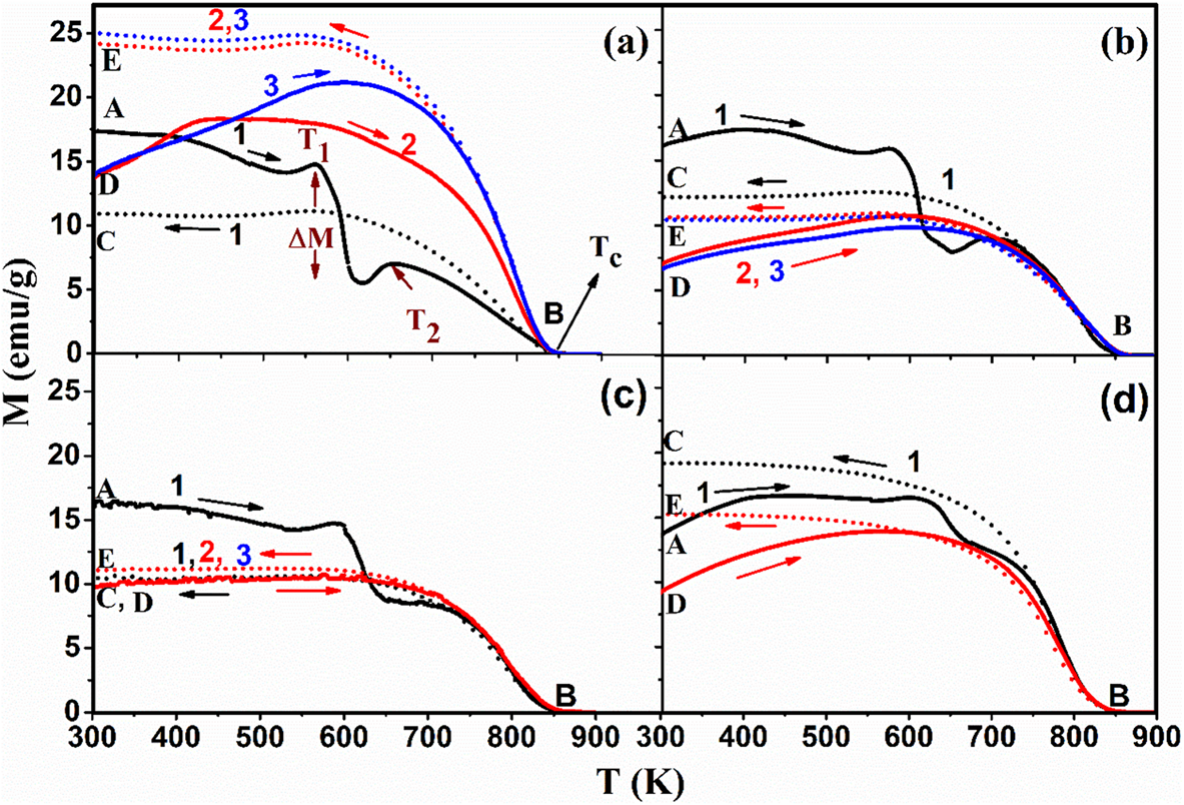
Change of magnetization (M) with temperature of pristine and Sn_*x*_Fe_3-2/3*x*_O_4_ nanoparticles of Sn^2+^ (*x*) amount **a** 0.000 (pristine Fe_3_O_4_), **b** 0.045, **c** 0.090, and **d** 0.150 respectively, for different heating–cooling cycles [for **a** and **b**, black indicates 1st; red, 2nd; blue, 3rd and for **c** and **d**, only 2-cycles are indicated] (magnetic field *H* = 200 Oe) (solid line, heating; dotted line, cooling)

**Fig. 2:**
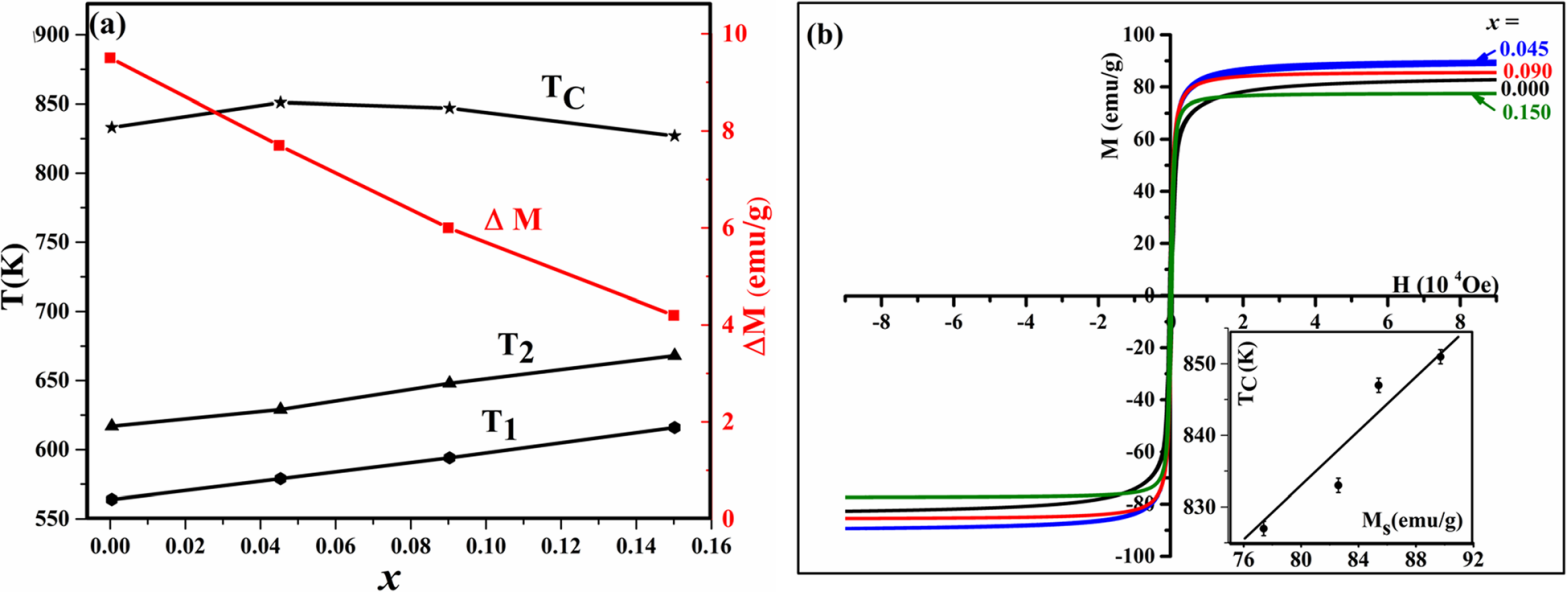
**a** *T*_1_, *T*_2_, Δ*M*, and *T*_c_ values obtained during 1st heating regime and **b** hysteresis loops for different amount of *x* for Sn_*x*_Fe_3-2/3*x*_O_4_ nanoparticles at 2 K (inset, the relation between Curie temperature and saturation magnetization)

Figure 1a–d shows the change of magnetization (*M*) as a function of temperature of the samples; pristine Fe_3_O_4_- and tin–doped Sn_*x*_Fe_3-2/3*x*_O_4_ nanoparticles with different amount of *x*. The samples were heated from 300 up to 900 K (Fig. 1 point A to B) and cooled back (point B to C) for the first heating–cooling cycle while applying an external magnetic field of 200 Oe. The heating–cooling cycle’s measurements as depicted from curves D to E were repeated under same magnetic field until stable magnetization data was reached. The pristine Fe_3_O_4_ nanoparticles (Fig. 1a) undergo heating–cooling cycle for five times. For clarity, we only present three cycles since after that there were no more changes in magnetization during heating–cooling process. The doped samples (Fig. 1b–d) were heated and cooled for three times only since there was no obvious change in M after the second cycle (two cycles are presented in the figures). Four obvious features were noticed where temperature is ranging from 300 to 900 K. First, there is a dip in magnetization of about 10 emu/g that occurred in the pristine sample (*x* = 0.000) between *T*_1_ (564 K) and *T*_2_ (655 K), while going from point A to B in the first heating–cooling cycle. This dip also occurred in the doped samples but with increased dipping temperatures (*T*_1_, *T*_2_) as *x* increases (Fig. 2a). This increase may be attributed to the increase in particle size due to Sn-doping as confirmed by HRTEM measurements shown in Fig. S1. To ensure that Sn^2+^ ions spread uniformly throughout the structure, an elemental mapping for the pure and Sn_*x*_Fe_3-2*x*/3_O_4_ doped sample with *x* = 0.150 (Figs. S2 and S3).

Similar dipping was also reported as mentioned above in oleate-capped magnetite nanoparticles with 20 nm in size which is attributed to the thermal decomposition of the capping ligands. Along with the decomposition, a reduction of Fe^3+^ to Fe^2+^ after heating was also observed using Raman and Mössbauer spectroscopy [14].

Interestingly, the dipping feature was not detected in the un-capped Fe_3_O_4_ sample reported by Kolen’ko et al. [14]. Although, there were no capping ligands used in our sample’s preparation, the surface of nanoparticles was influenced by oxidation either to maghemite (γ-Fe_2_O_3_) or Sn^2+^-related oxides, both of which could act as a capping layer. Consequently, the dipping of M in the first heating–cooling cycles indicates that there was a thermal decomposition of the oxidized layer on the surface of these nanoparticles (i.e., a reduction of Fe^3+^ and Sn^2+^, Sn ^4+^ ions).This decomposition will take place at lower temperature for smaller particles due to their larger specific surface area. This explanation is supported by a previously reported reduction of amorphous γ-Fe_2_O_3_ nanoparticles in an evacuated environment at 523 K [20]. The second observed feature is related to the *M* dipping amplitude (labeled as Δ*M* in Fig. 1a). Δ*M* decreases as the amount of Sn^2+^ increases (Fig. 2a) due to decrease in the amount of γ-Fe_2_O_3_ caused by doping process [11, 16].

**Fig. 3:**
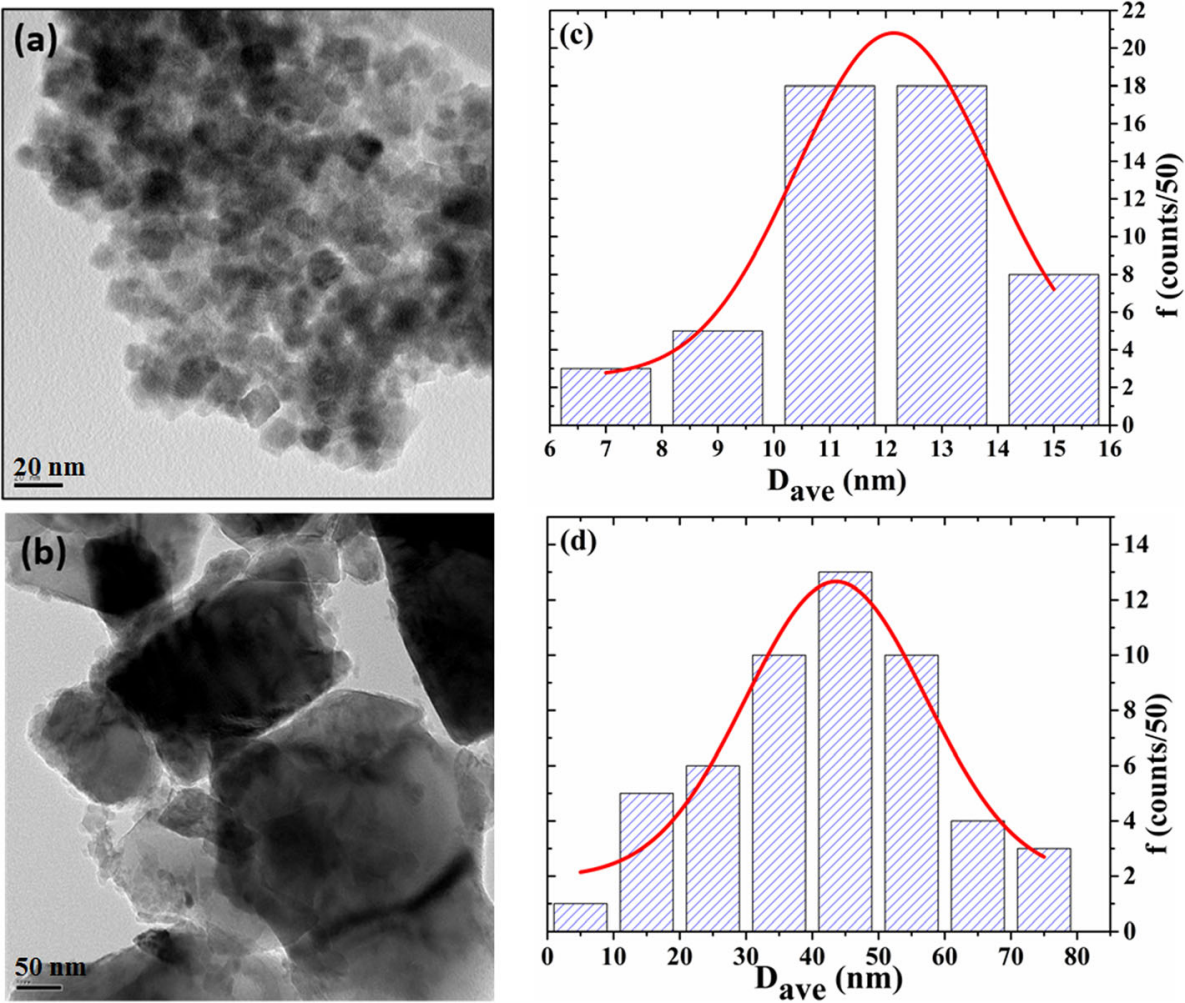
TEM image and size distribution histogram of prepared Fe_3_O_4_ nanoparticles **a**, **c** before annealing and **b**, **d** after heating to 900 K (the red solid lines at **c** and **d** are the normal fitting)

The third feature is that the heating–cooling curves are irreversible (i.e., *M* curves during heating are different from cooling). This is related to the blocking features since after heating there is an increase in the particle size confirmed by TEM images (Fig. 3). The increase in particle size will increase the magnetocrystalline anisotropic energy (*E*_*A*_) of a single domain particle according to Wolfarth model as shown below.

1 $$ {E}_A= KV\ {\mathit{\sin}}^2\theta $$

where *K* is the magnetocrystalline anisotropy constant, *V* is the volume of the nanoparticle, and *θ* is the angle between the magnetization direction and the easy axis of magnetization of the nanoparticles [21, 22]. Hence, more thermal energy is needed to overcome the magnetic anisotrpic energy and randomize the magnetic spins. The randomly oriented spins as a result of heating will start to be affected by the applied magnetic field at a certain temperature via cooling. When the temperature reaches *T*_2_, these aligned spins will be blocked attaining high constant magnetization while approaching room temperature (detailed explanation is in the “The Origin of Divergence in Heating–Cooling Graph” section). The fourth feature is the dependence of Curie temperature (*T*_C_) on the amount of Sn^2+^ doped as shown in Fig. 2a and this is related to the effect of Sn^2+^ ions on the saturation magnetization (M_s_) as shown in Fig. 2b. Hence, it is anticipated that as *M*_s_ increases, *T*_C_ will increase as shown in the inset of Fig. 2b, which is in good agreement to previous reports [11, 16]. All the four aforementioned features suggest a strategy for tuning of Fe_3_O_4_ nanoparticles magnetization, dipping temperature/amplitude, irreversibility, and Curie point by Sn^2+^ doping.

### Characterization of the Heated Samples

**Fig. 4:**
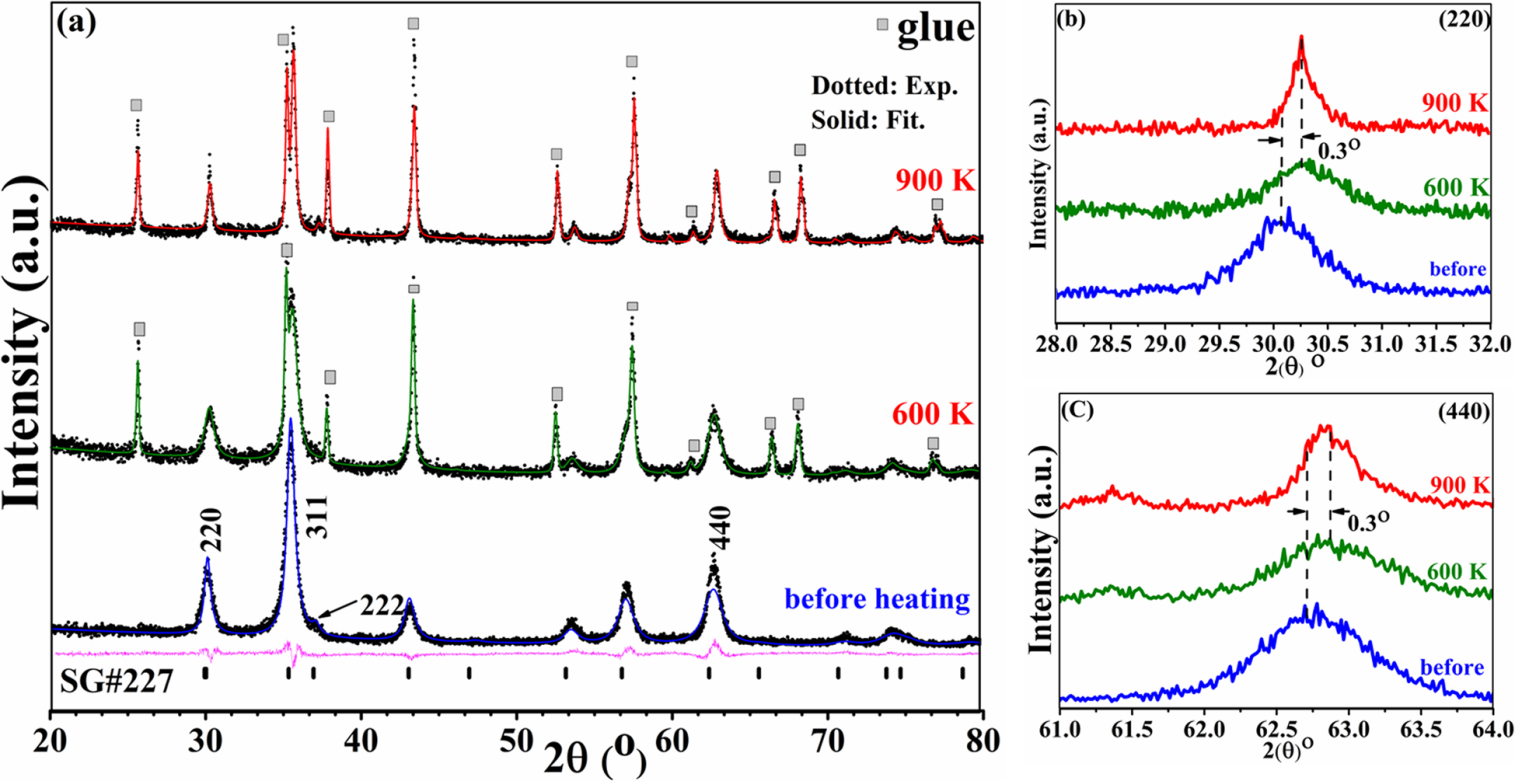
**a** XRD patterns for pristine Fe_3_O_4_ before heating and after heating to 600 K (green), 900 K (red) (black dotted line, experimental data; solid line, fitted; magenta, difference; pars, SG #227 phase) (the small filled squares represent the peaks for the glue used for high-temperature VSM measurements), **b** enlarge pattern for (220) peak, and **c** enlarge pattern for (440) peak

Although the results of the pristine sample heated to 900 K were obtained and discussed, in order to investigate the origin of the first dipping temperature (*T*_1_), additional structural and magnetic measurements were also carried out for the same sample after in situ heating at high-temperature VSM measurements up to 600 K. Figure 4a shows XRD patterns and their Rietveld refinements for the pristine sample before heating, after heating via high-temperature VSM measurements to 600 K and 900 K. The XRD peaks for the cement (glue) used to fix the sample on the heating stick for high-temperature VSM measurements are represented by small filled squares as a reference. Before heating, the pattern is indexed to the spinel related structure (SG# 227). There is an overlap between the 311 and 222 peaks, which normally appear at 2θ equal to 35° and 37° respectively. This is an indication of the existence of γ-Fe_2_O_3_ phase, since it has the same spinel structure of magnetite but with a smaller lattice parameter. This overlap disappears after heating to 600 K which indicates a decrease or inhibition of γ-Fe_2_O_3_ phase due to a reduction of Fe^3+^ to Fe^2+^ (neglecting the peaks topped with the square at about 35° which is referred to the glue). Moreover, since the (220) and (440) peaks appear at about 30° and 62°, respectively, are related solely to ferric oxides without the glue [23], we indicate in Fig. 4b and c enlarge pattern of these peaks. After heating to 600 K, both peaks undergo a shift to a higher reflection angle by about 0.3° which is an indication of a decrease in the (d) spacing values. This decrease is normally associated with the high-temperature annealing of oxide nanoparticles which often results in solvent removal and annihilation of defects and thus leads to a decrease in the values of lattice parameter [14]. The full width half maximum of both peaks decreases as a result of the crystallinity improvement and an increase in the crystallite size according to the Scherrer equation. The shape of the peaks changes from symmetric to asymmetric with steeper low-angle side. As mentioned above, both magnetite and maghemite phases have the same spinel structure but with slightly larger lattice parameter for magnetite (lower reflection angle); the asymmetry indicates an increase in the magnetite phase at 30.3° with the lower angle peak compared to maghemite at 30.5°. This reduction of γ-Fe_2_O_3_ phase will increase the value of M at T_1_ since magnetite has larger saturated magnetization and it is non-repeatable process that occurring at the first heating–cooling cycle which explains the change in the *M*-*T* curve for the subsequent heating–cooling cycles. After heating to 900 K, the peaks sharpen while remaining at the same angle indicating a more increase in crystallite size confirmed by TEM images (Fig. 3) (from 12 nm to 30 nm). This sharpness is reflected in *M*-*T* curve as an increase in *M* at *T*_2_.

**Fig. 5:**
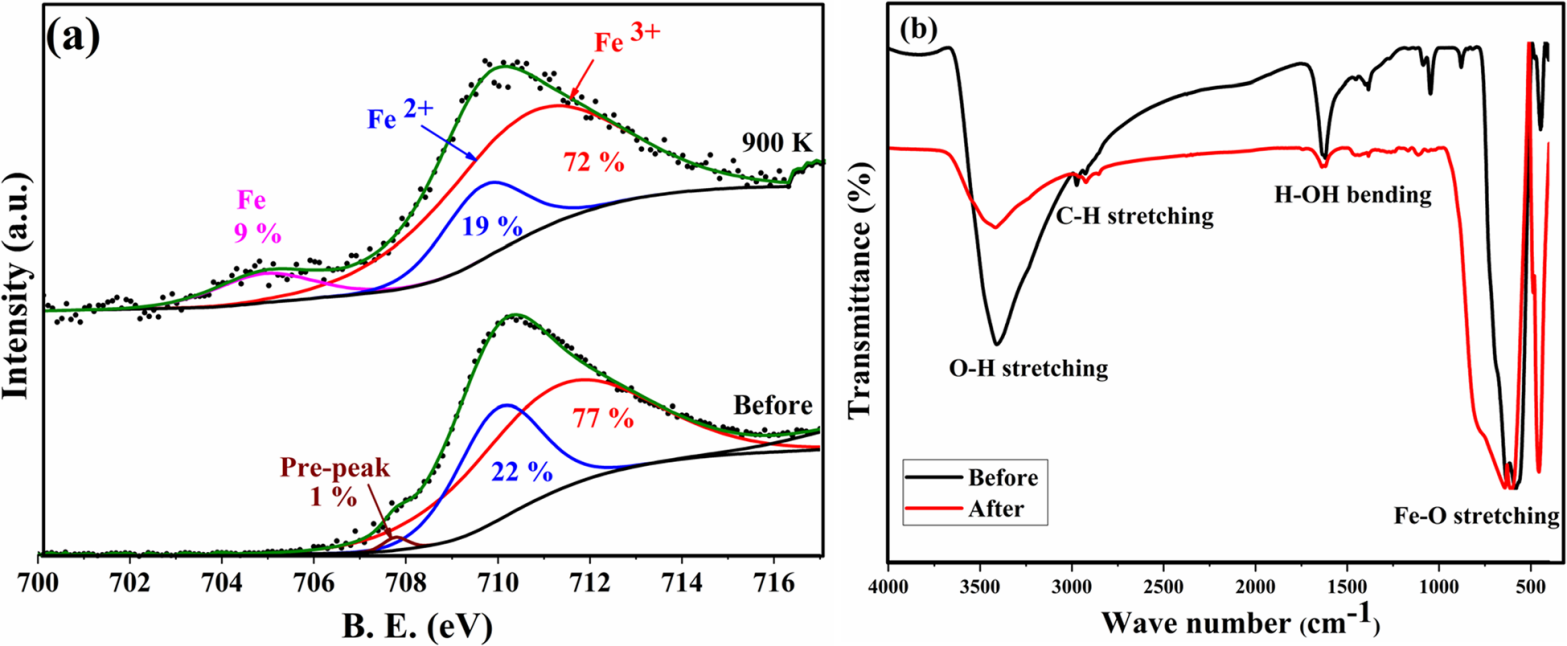
**a** Deconvoluted high-resolution XPS spectra of Fe 2p_3/2_ recorded from pristine Fe_3_O_4_ sample before and after heating to 900 K (red, Fe^3+^; blue, Fe^2+^; magenta, Fe metallic tail). **b** FTIR spectra (transmission vs. wavenumbers) of Fe_3_O_4_ nanoparticles before and after heating to 900 K

Since the asymmetric feature of the two peaks (220) and (440) is not solely providing solid evidence to distinguish between the two-spinel magnetite and maghemite phases using XRD. Thus, the reduction or inhibition of γ-Fe_2_O_3_ phase at high annealing temperatures was confirmed by XPS measurements. Figure 5a shows the XPS core-level ionization Fe 2p_3/2_ spectra obtained from pristine sample before and after heating to 900 K. Two components can be found from the deconvoluted Fe 2p_3/2_ peak at binding energies of 709 eV and 711 eV representing Fe^2+^ (22%) and Fe^3+^ (77%) states, respectively, with a pre-peak low energy tail at 708 eV [24, 25]. Upon heating at 900 K along with the reduction in binding energy of the two components, a certain amount of Fe^3+^ (72%) states transforms to Fe^2+^ (19%) and the metallic Fe (9%)—component depicted at 705 eV—as a reflection of the reduction of γ-Fe_2_O_3_ phase.

The FTIR spectra of pristine Fe_3_O_4_ nanoparticles before and after heating to 900 K are shown at Fig. 5b. The strong peaks at 583 cm^−1^ and 634 cm^−1^ are assigned, as indicated in the figure, to the stretching of Fe-O bonds. After heating the sample, these peaks broadened and shifted to higher frequencies indicating a strengthening in the Fe-O bonds due to the crystallinity improvements and the increase in crystallite size proved using XRD measurements. The peaks between 1402 cm^−1^ and 878 cm^−1^ are related to adsorbates features [26–28] and disappeared after heating at 900 K. The peaks at 3413 cm^−1^ and 2974 cm^−1^ are related to the stretching bonds coming from the environmental OH^−^ and CO_2_ groups, respectively [27]. The intensity of these peaks decreases by heating which is accepted due to sintering process. The peak at 1619 cm^−1^ is related to the bending of the bond related to the hydroxide group coming from the atmosphere and its intensity also decreases by heating.

**Fig. 6:**
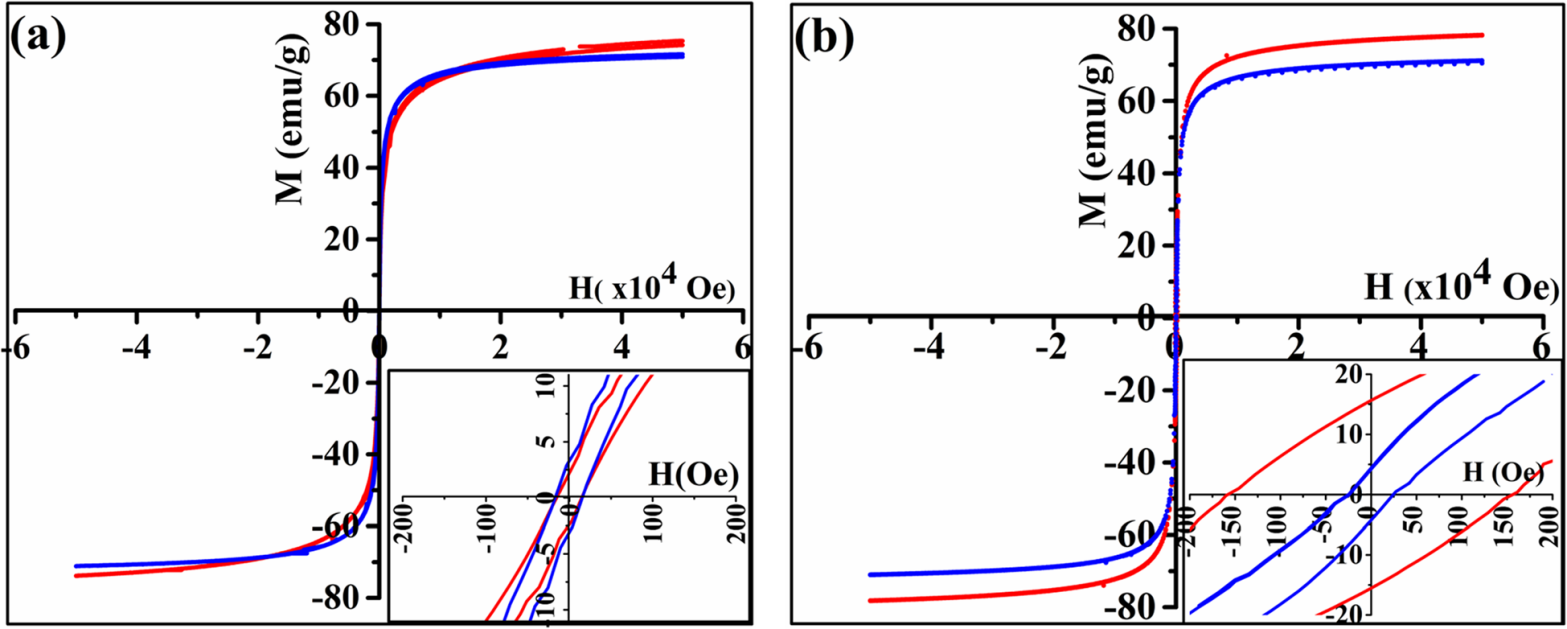
Hysteresis loops for pristine Fe_3_O_4_ nanoparticles before (blue) and after heating (red) up to **a** 600 K and **b** 900 K (insets show magnetization at low magnetic field)

Consequently, the change in magnetization due to the reduction process at *T*_1_ and sintering process at *T*_2_ causes the observed dipping in magnetization. The hysteresis loops for the pristine sample before and after heating up to both 600 K and 900 K (Fig. 6) indicate a little increase in *M* after heating which is supporting the reduction of Fe^3+^ ions at *T*_1_. The remanence and coercivity (inset of Fig. 6) were increased after heating to 900 K, while they did not change after heating to 600 K, which verifies that the sintering process takes place at *T*_2_, hence confirming what was found from XRD and FTIR measurements.

### The Origin of Divergence in Heating–Cooling Graph

**Fig. 7:**
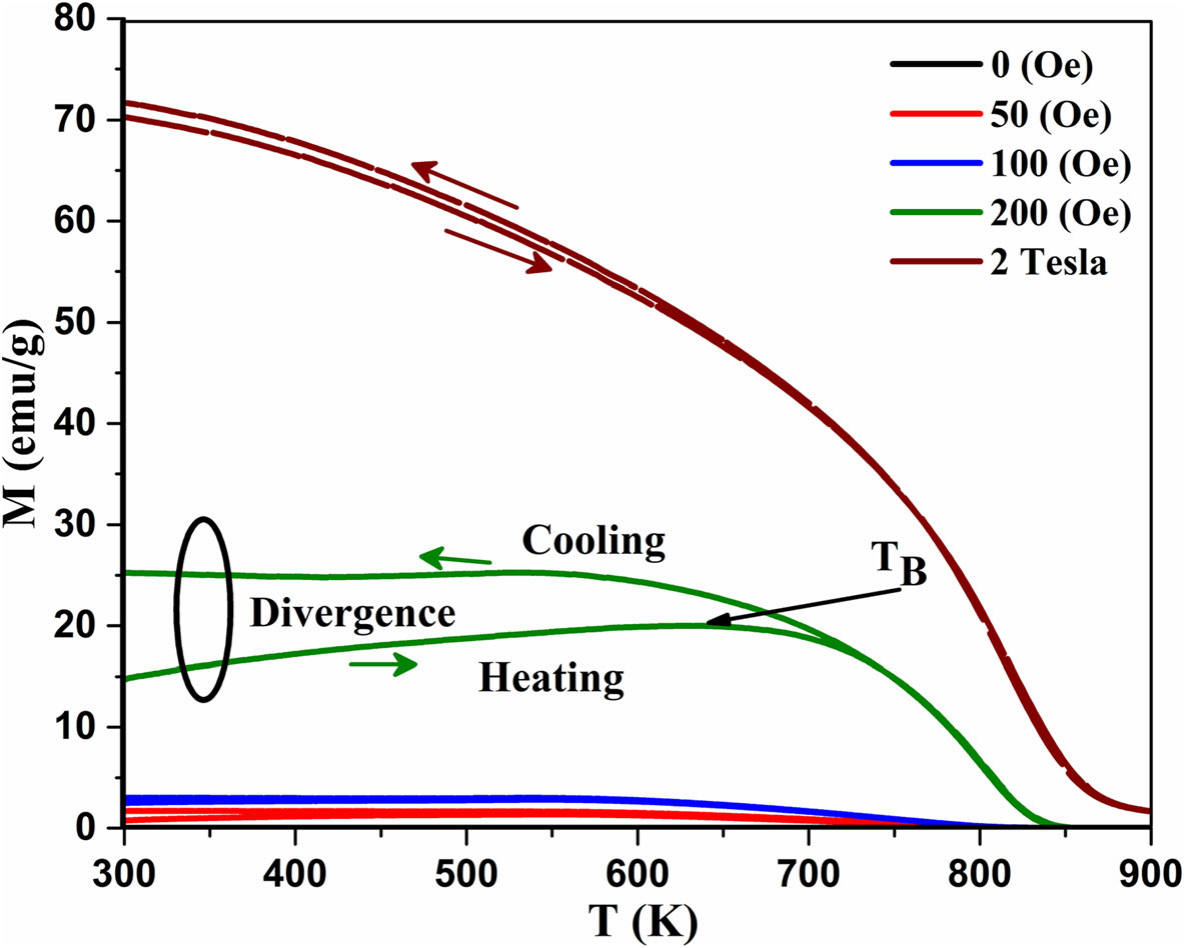
Change of magnetization (*M*) with temperature for pristine Fe_3_O_4_ nanoparticles at different external magnetic field (*H*). At *H* = 200 Oe, blocking temperature *T*_*B*_ and magnetic divergence (labeled by circular ring) between the heating and cooling curves can be seen clearly

To investigate the origin of the observed divergence in M while heating and cooling (Fig. 1) and its relationship to the blocking temperatures, more measurements on the pristine sample subjected to different external magnetic fields were performed, while heating and cooling, as shown in Fig. 7. It can be seen clearly that the divergence (labeled as a circular ring) disappeared when the measurements were collected while applying high magnetic field of 2 T (i.e., this divergence simplifies the identification of the blocking temperatures of these nanoparticles at external magnetic fields of 200 Oe).

**Fig. 8:**
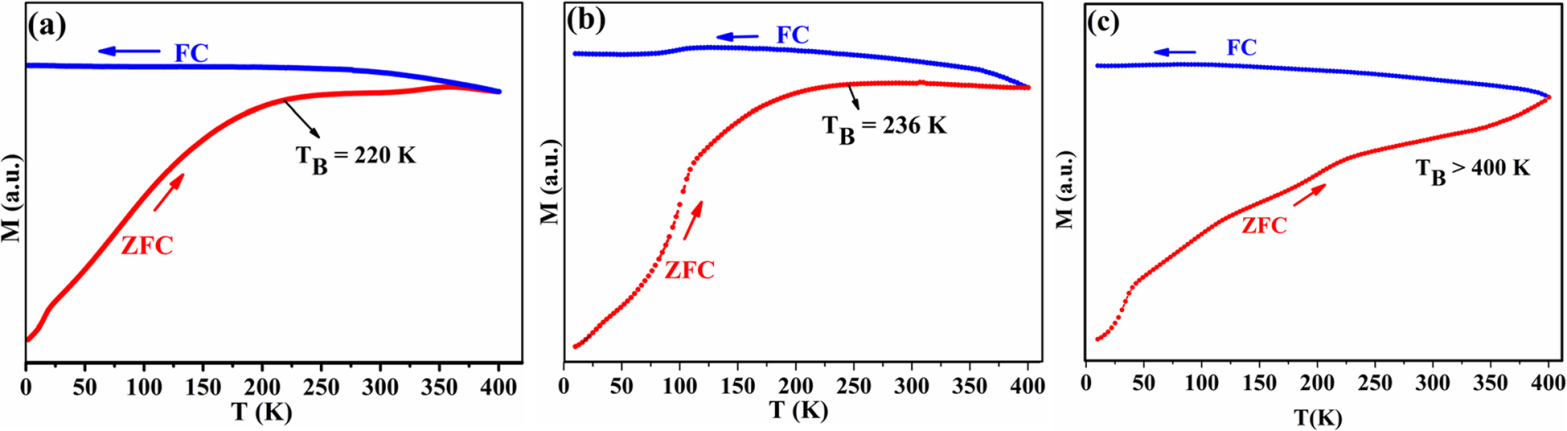
ZFC-FC (*M*-*T*) curves at low temperatures (*H* = 200 Oe) for the pristine Fe_3_O_4_ **a** before heating **b** Pristine Fe_3_O_4_ with cement used as glue after heating up to 600 K and **c** 900 K

Based on that, additional low temperature VSM measurements (2–400 K) using the zero field cooling–field cooling (ZFC-FC) protocols with external magnetic field of 200 Oe were made for the pristine sample after subjected to high-temperature VSM measurements up to 600 K and 900 K and compared with the same sample before heating (Fig. 8).

**Fig. 9:**
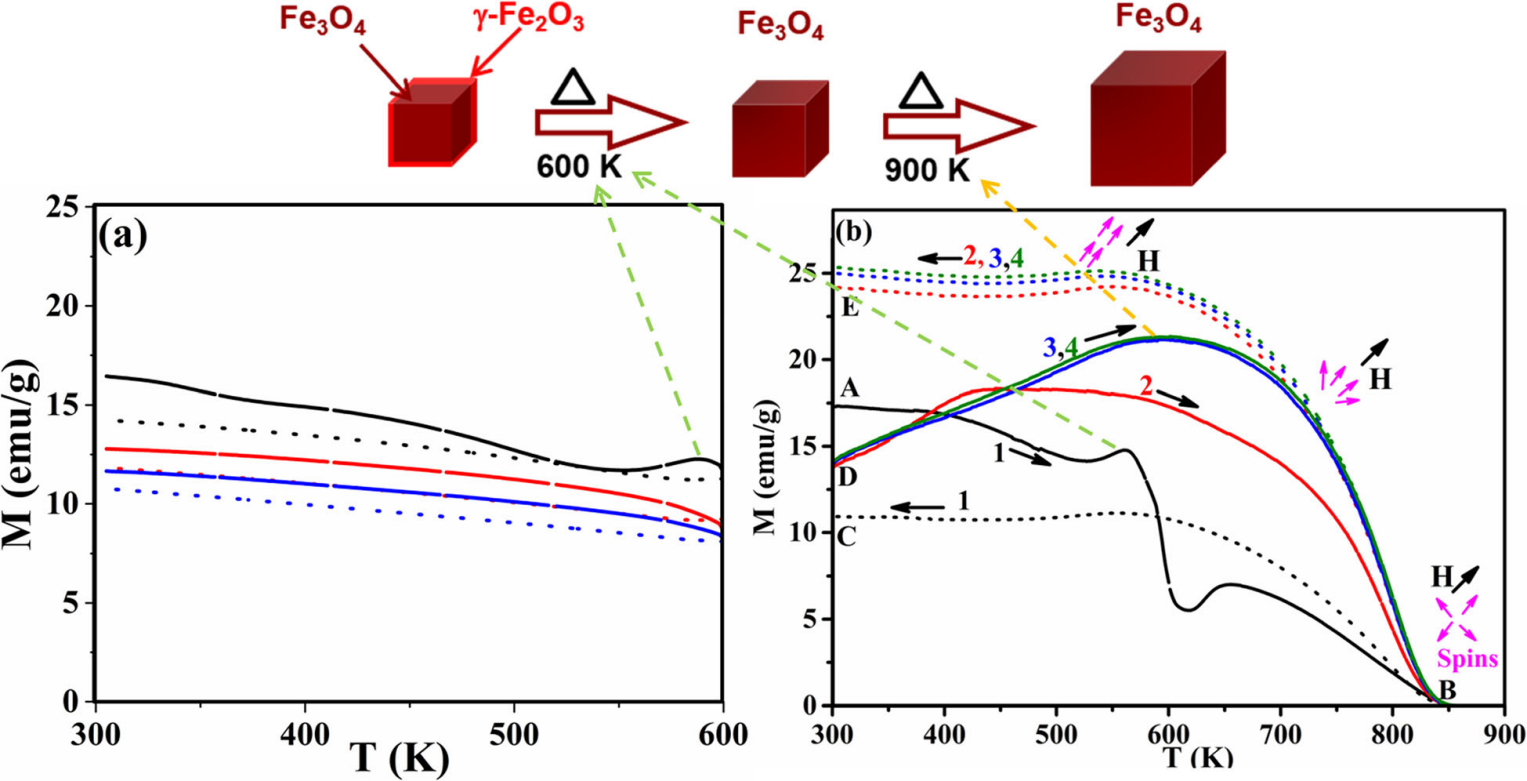
Change of magnetization (*M*) with temperature (*T*) for pristine Fe_3_O_4_ while heating up to **a** 600 K and **b** 900 K using magnetic field of 200 Oe at three heating–cooling cycles. The schematic diagram on top of the figure represents the change of morphology of the NPs as temperature increases from 300 to 900 K (Initially, the Fe_3_O_4_ NPs are covered with a thin surface layer of γ-Fe_2_O_3_ which acts as a shell. Upon heating to 600 K, the γ-Fe_2_O_3_ annihilation takes place and NPs agglomeration start to occur till 900 K, magenta arrows represent spin’s orientation)

The blocking temperature for the sample heated to 900 K was higher than that of the sample heated to 600 K and to the non-heated sample. This was expected since the sample heated to 600 K shows a very small divergence in heating/cooling regime (Fig. 9a). This reinforces that at 600 K, there is a reduction from Fe^3+^ to Fe^2+^ without any increase neither in particle size nor in blocking temperature. Hence, we conclude that the first dipping temperature is referred to the reduction while the second temperature referred to the increase in particle size as shown schematically in Fig. 9. The same feature (increasing *M* while cooling) is obvious for the sample with *x* = 0.150 from the first heating–cooling cycle (Fig. 1d), which proves that doping with this amount of Sn will give the same thermomagnetic trend and will block the spins at higher temperatures during cooling regime. This makes Sn_*x*_Fe_3-2/3*x*_O_4_ with *x* = 0.150 be more practical and applicable when needed to be used at high temperatures. It should be mentioned that the divergence feature in oleate-capped Fe_3_O_4_ was previously reported by Kolen’ko et al. and attributed to the existence of γ-Fe_2_O_3_ in their sample. However, this is not the case since it is revealed to be related to the externally applied magnetic field as explained and depicted in Fig. 7. Hence, during heating up to the new blocking temperature (*T*_2_), the magnetization increased because of the thermal excitations of the blocked magnetic moments. However, while cooling down to the blocking temperature again the spins blocked at high magnetization and the thermal energy could not overcome the magnetic energy caused by the applied magnetic field as indicated by magenta arrows at Fig. 9.

### The Surface Effect

**Fig. 10:**
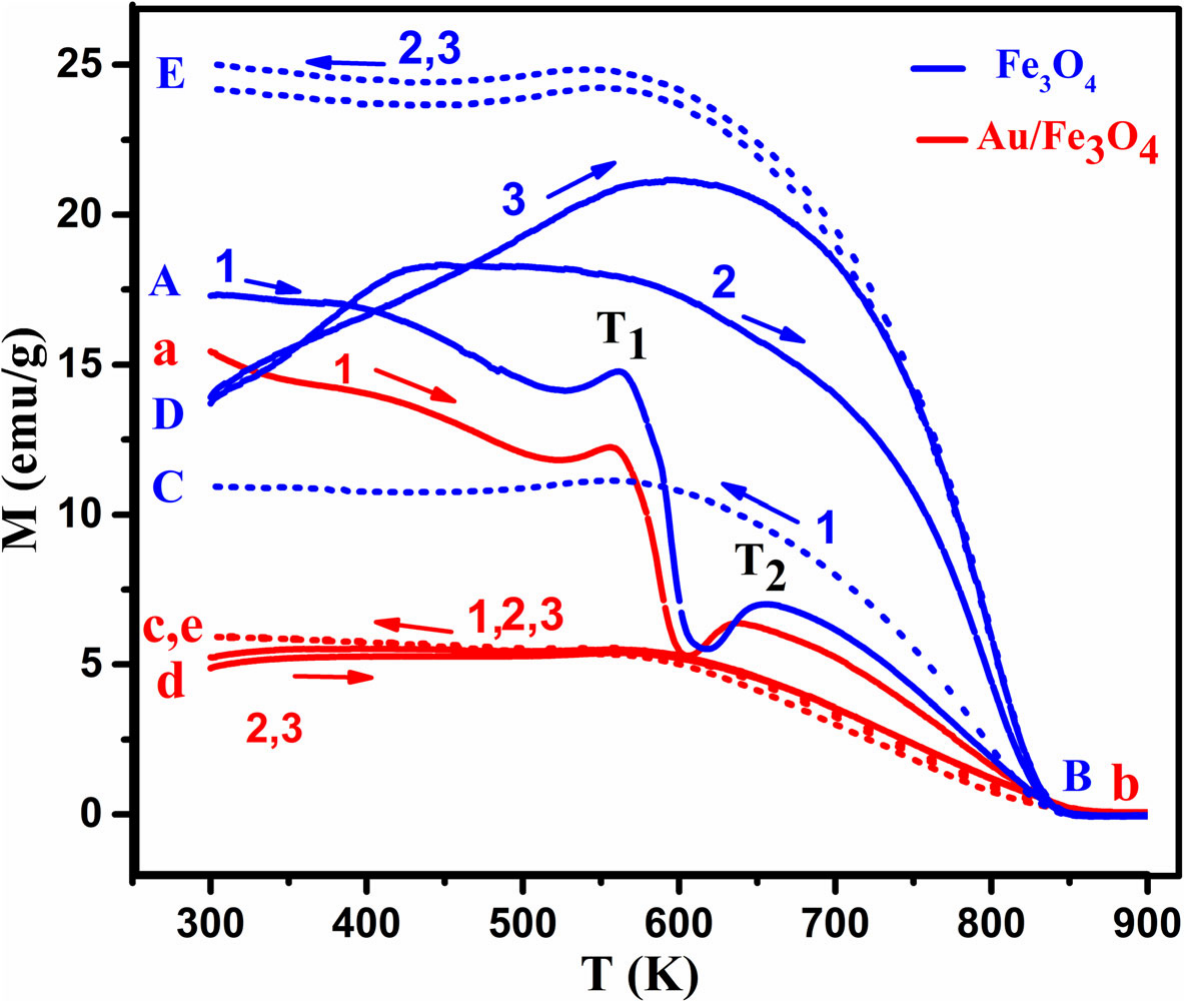
Change of magnetization (*M*) with temperature of pristine Fe_3_O_4_ (blue, capital letters) nanoparticles and Au/Fe_3_O_4_ (red, small letters) for three indicated sequential heating–cooling cycles (magnetic field *H* = 200 Oe) (solid line, heating; dotted line, cooling)

In order to investigate the effect of agglomeration of these nanoparticles in magnetization, a small amount of the pristine Fe_3_O_4_ sample was covered with a thin layer of Au (~ 2 nm) using the evaporation technique. The *M*-*T* graphs for the pristine Fe_3_O_4_ nanoparticles with and without gold after heating up to 900 K and cooling back for three cycles are shown in Fig. 10.

It can be noticed that the dipping amplitude (Δ*M*) decreases for the particles covered with gold similar to the behavior observed by doping with Sn^2+^ and can be attributed to the decrease in the oxidation reaction (i.e. in the amount of γ-Fe_2_O_3_ phase) by coating with Au on the surface of these nanoparticles. For the second dipping temperature (*T*_2_), there are two observations. First, like the pristine nanoparticles, there is an increase in the magnetization at *T*_2_. At this temperature, the thermal energy will unblock the spins of these nanoparticles and align them in the direction of the magnetic field. However, *T*_2_ value decreases for the Au/Fe_3_O_4_ nanoparticles, since now the interparticle interactions will be less and consequently reduce the energy needed to unblock the spins.

**Fig. 11:**
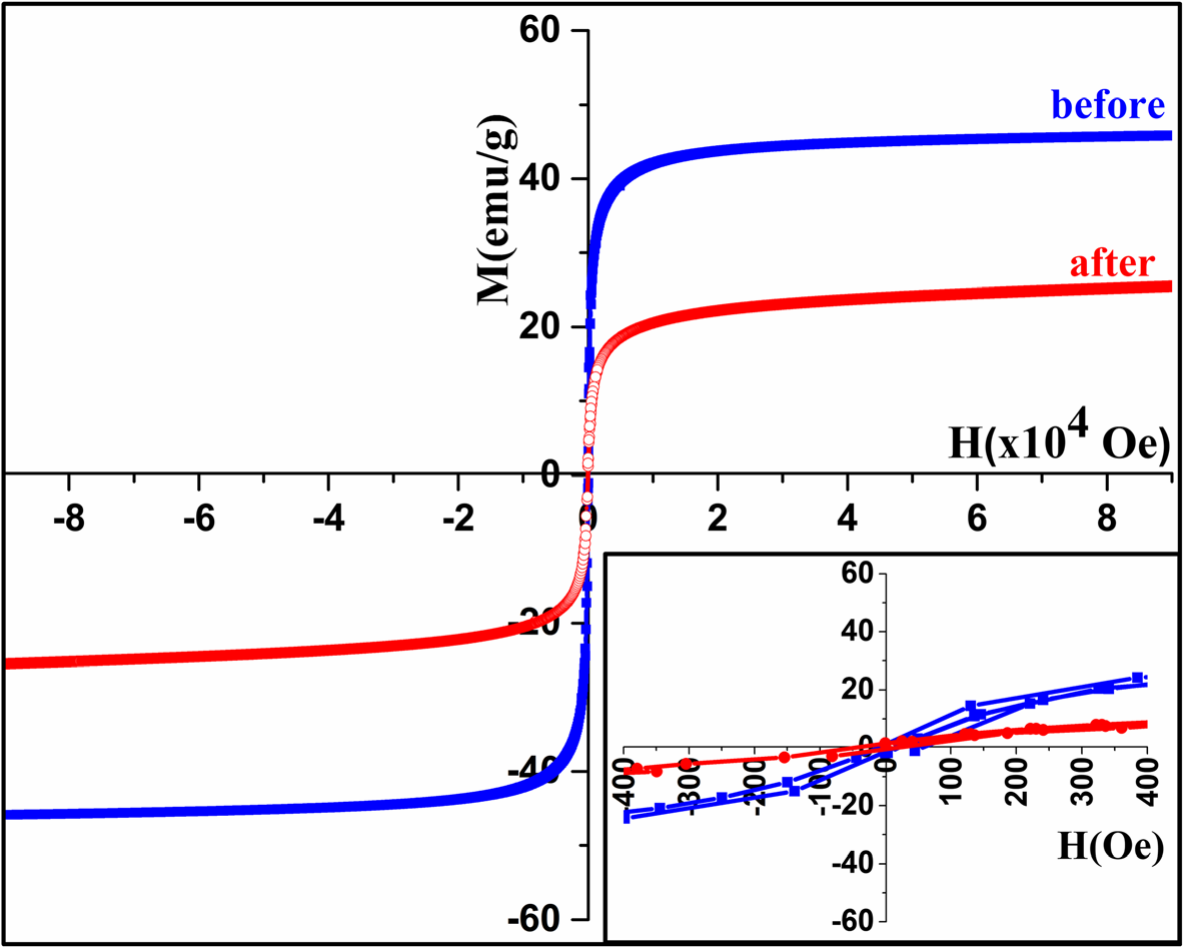
Hysteresis loops for Au/Fe_3_O_4_ nanoparticles before and after heating to 900 K (inset at low magnetic field) (blue, before heating; red, after heating) (inset shows the hysteresis loops at low fields)

Since Au reduces the agglomeration of these nanoparticles, the divergence in heating–cooling cycles that appeared for the pristine nanoparticles after the second cycle is very small. The hysteresis loops made for Au/Fe_3_O_4_ sample before and after heating (Fig. 11) shows a decrease in M after heating which may be referred to the diamagnetic effect of Au. The coercivity and remanence did not change which proves that there is no agglomeration, change in particle size or on the crystallinity of these nanoparticles after coating with gold.

### Theoretical Explanation

**Fig. 12:**
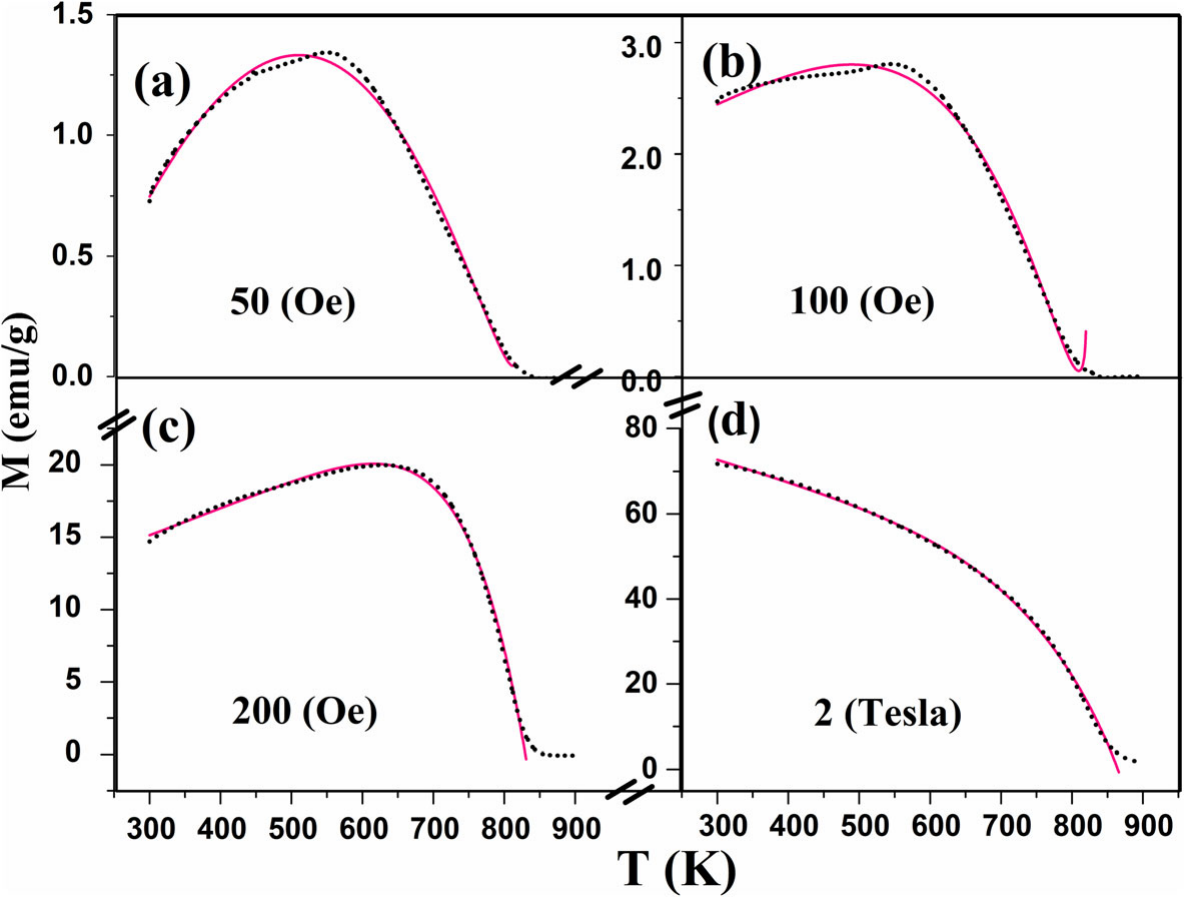
Change of magnetization (*M*) with temperature during heating (after 3rd cycle) of the heated pristine Fe_3_O_4_ nanoparticles while applying different magnetic field *H* of **a** 50 (Oe), **b** 100 (Oe), **c** 200 (Oe), and **d** 2 T (black dotted, experimental; pink solid, fitted using Eq. 4)

**Fig. 13:**
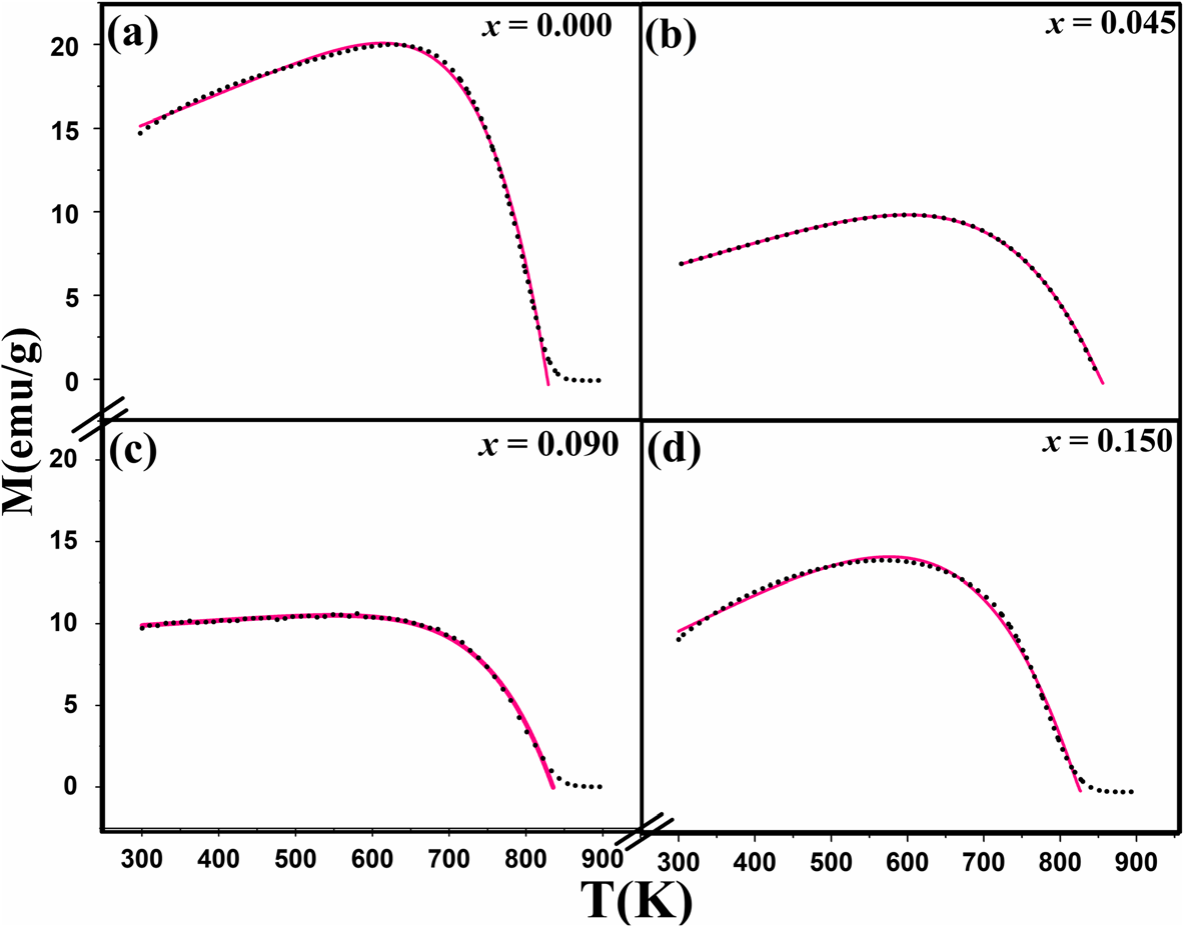
Change of magnetization (*M*) with temperature during heating (the 3rd cycles) of the heated Sn_*x*_Fe_3-2/3*x*_O_4_ nanoparticles with different amount of the indicated *x* (0.000, 0.045, 0.090, 0.150) (*H* = 200 Oe) (black dotted, experimental; pink solid, fitted)

It is imperative to discuss two challenges faced while trying to understand the observed features of high-temperature NPs magnetization after reaching stable repeatable measurements (≈ 3rd cycles). The first is due to the deviation of the Bloch law normally used for the bulk to explain the observed change of in saturation magnetization with temperature for magnetic nanoparticles [29–31]. In this regard, many efforts have been made to modify Bloch law such as that reported by Kodama et al. [32]. They started with Bloch formula:

2 $$ \mathrm{M}={\mathrm{M}}_0{\left[1-\upgamma \left(\frac{\mathrm{T}}{{\mathrm{T}}_{\mathrm{C}}}\right)\right]}^{\upbeta} $$

and allowing the parameters *γ* and *β*—equal 1 and 3/2 for the bulk material, respectively—to change. Consequently, the value of *β* was found to lay between 3/2 and 2 for NPs. The increase in *β* value compared to that of the bulk is related to the collective thermal excitations of the ordered spin which produces an energy gap (Δ*E*) between the ordered and disordered spins. This energy gap will reduce the spontaneous magnetization by an amount proportional to exp (− Δ*E*/*k*_*B*_*T*). Hence, Kodama et al. suggested to use the same value of *β* for the bulk (3/2) but by adding exp (− Δ*E*/*k*_*B*_*T*) to Eq. 2. The second challenge is that our measurements were done in low magnetic fields and cannot be fitted with Bloch law alone since the spins are not saturated and the energy gap (Δ*E*) will be affected by the magnetic field leading to change the measured magnetization. Motivated by the aforementioned challenges and in order to fit and justify our observed *M*-*T* graphs at different magnetic fields and different Sn^2+^ concentrations, a simple phenomenological expression that combines both the modified Bloch law and Curie–Weiss law was introduced. This justification is based on a core-shell structure model for these nanoparticles [29]. Hence, we assume that each nanoparticle is composed of a core with saturated spins and a bulk like interchange interactions surrounded by a shell with randomly oriented spins. In the core, the magnetization is given by:

3 $$ {\mathrm{M}}_{\mathrm{H}-\mathrm{core}}={\mathrm{M}}_{\mathrm{H}}{\left[1-\upgamma \left(\frac{\mathrm{T}}{{\mathrm{T}}_{\mathrm{C}}}\right)\right]}^{\upbeta} $$

which is the same modified Bloch law in Eq. 2 but by replacing Mo with *M*_*H*-_ where the value of *M* at 300 K and at certain magnetic field. For the shell, there is no interchange interactions between the magnetic spins—like paramagnetic materials—and the *M*-*T* relation in this part (*M*_*H*-Shell_) will obey Curie–Weiss law as *M*_*H*-Shell_ = *C*/(*T* − *T*_*C*_), where *C* is the Curie constant. Hence, the deviation of our *M*-*T* curves from the modified Bloch law is related to the shell effect that decreases the magnetization and will disappear at high magnetic fields and high temperatures. The measured magnetization at each temperature (*M*_exp_) will be the total contribution of both the core and the shell parts. The best fit for the experimental magnetization (*M*) of the pristine sample with the magnetic field (*H*) (Fig. 12) and for *M* of the Sn_*x*_Fe_3-2/3*x*_O_4_ with *x* (Fig. 13) was reached by applying the formula

4 $$ {\mathrm{M}}_{\mathrm{exp}}={\mathrm{M}}_{\mathrm{H}}{\left[1-\upgamma \left(\frac{\mathrm{T}}{{\mathrm{T}}_{\mathrm{C}}}\right)\right]}^{\upbeta}-\upalpha {\left(\mathrm{T}-{\mathrm{T}}_{\mathrm{C}}\right)}^{\updelta} $$

where *α*, *β*, *δ* , *γ*, *M*_*H*_, and *T*_*C*_ are parameters to be derived from the fitting. The second term will be positive for *T* < *T*_*C*_. We free the power (*δ*) in the second part of Eq. 4 to see how it can affect the quality of our fitting. In order to verify our results, we tested the modified Bloch law proposed by Kodama et al. for the pure sample at high magnetic field of 2 T and the value of *β* was 2.6. This value is within the suggested range for this size of nanoparticles [32].

**Fig. 14:**
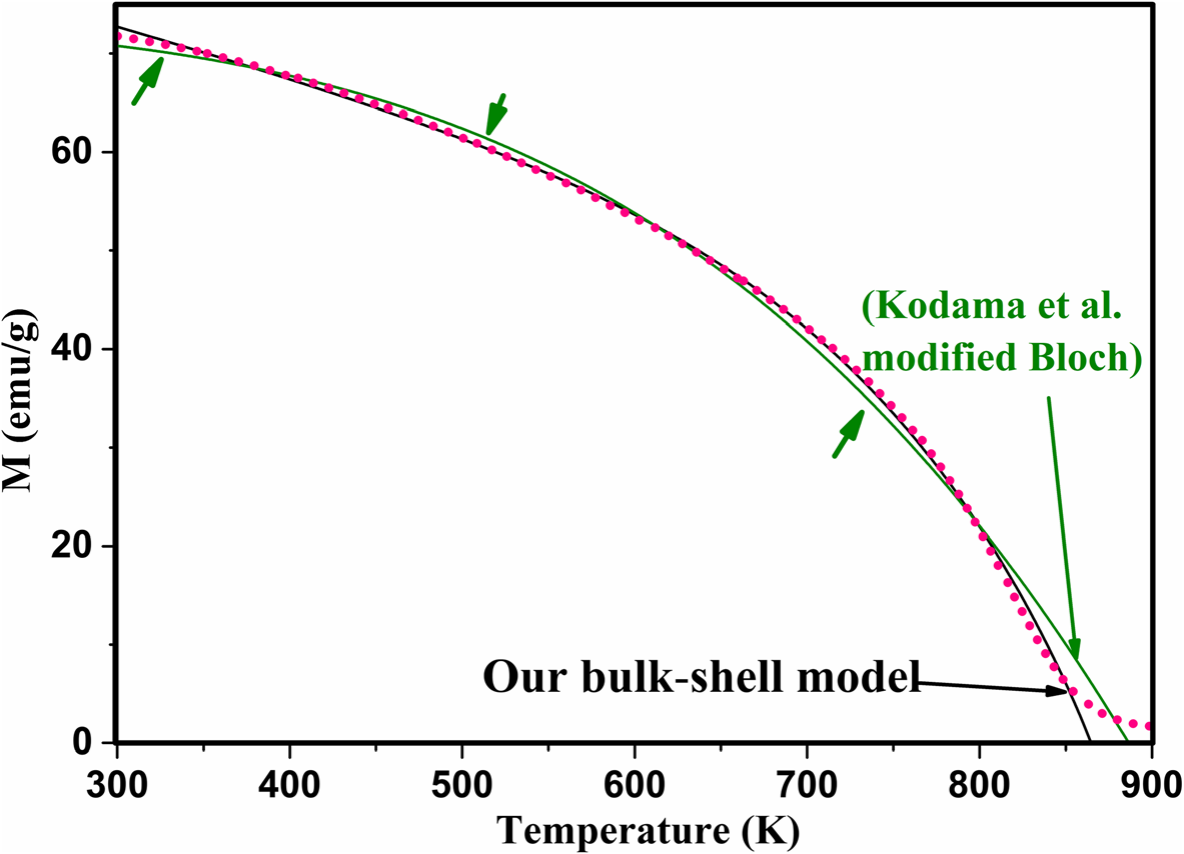
Change of magnetization (*M*) with temperature during heating for the 3rd cycle of the heated pristine Fe_3_O_4_ nanoparticles while applying a magnetic field *H* = 2 (Tesla) (pink dotted, experimental; solid, fitted using the new bulk-shell expression (black) and the modified Bloch law proposed by Kodama et al. (green)). Green arrows indicate the temperatures where the modified Bloch law proposed by Kodama et al. failed to fully fit the experimental data

However, as can be seen in Fig. 14, fitting our *M*-*T* curves with the core-shell-related expression (Eq. 4) is better than the suggested modified Bloch law specially at high temperatures and low magnetic fields (i.e., for unsaturated magnetic spins).

**Fig. 15:**
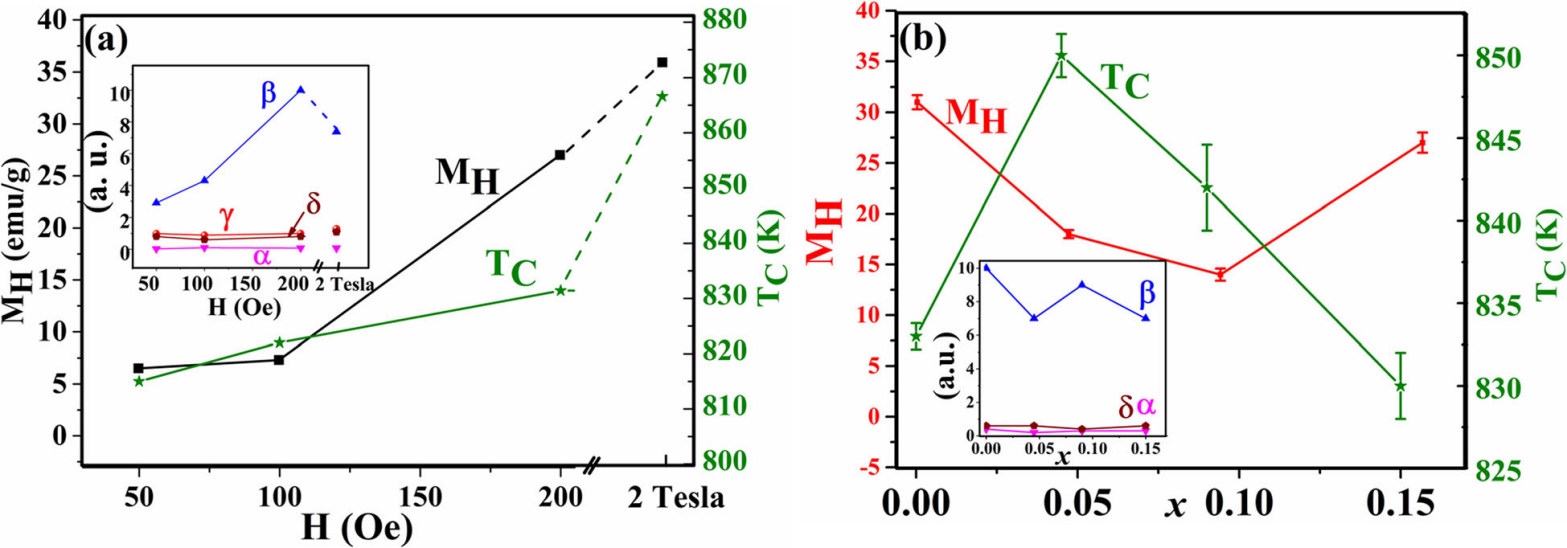
**a** Change for the pristine Fe_3_O_4_ of *M*_*H*_ (left) and *T*_*C*_ (right) with the applied external magnetic field H and (the insets show the change in different parameters *α* (purple), *β* (blue), and *δ* (brown) in both cases and with *γ* (red) with respect to external magnetic field) **b** for Sn_*x*_Fe_3-2/3*x*_O_4_ samples as a function of *x* taken at *H* = 200 Oe (the insets show the change in different parameters *α* (purple), *β* (blue), and *δ* (brown) in both cases and with *γ* (red) with respect to *x*)

The change of the parameters in Eq. 4 with the applied magnetic field for the pristine Fe_3_O_4_ nanoparticles is shown in Fig. 15a. It can be noticed that *M*_*H*_ increases as it is expected with the increase in the magnetic field. The values of *γ* and *δ* ≈ 1 and do not change with the applied field as they are depending only as mentioned above on the material structure and the particle size.

The (*α*) parameter is a very small constant. It turns to negative sign for higher field which is reasonable since the high field will saturate the spins at the shell and the paramagnetic effect will be small. The *β* values fluctuated ranging from 3 to 10 with the magnetic field which is different than the obtained power for nanoparticles using modified Bloch law. This is acceptable since we use *M*_*H*_ at 300 K instead of the saturated *M*_*s*_ in Bloch law. The *T*_*C*_ values, which are the same as what founded experimentally at 200 Oe in Fig. 2a, also changes with the applied field—a characteristic feature previously reported for magnetic nanoparticles [33].

Figure 15b shows the change of these parameters with the amount of Sn^2+^ (*x*). *M*_*H*_ does not behave like the previously found saturation magnetization (*M*_*s*_) (Fig. 2b) since *M*_*H*_ is related to the magnetic field and the size of these nanoparticles. It is accepted that *M*_*H*_ is larger for the pristine nanoparticles because of the reduction of γ-Fe_2_O_3_ phase and the sintering processes that took place during the previous heating–cooling cycles, which increased the saturated magnetization. For the Sn^2+^-doped sample, *M*_*H*_ decreases since the existence of Sn^2+^ at the surface which can prevent the agglomeration process and the crystal growth (can be verified using TEM or XRD). The value of *M*_*H*_ for *x* = 0.045 is larger than for *x* = 0.090 which is consistence with the larger value of *M*_*s*_ for this sample. Interestingly, for the larger NPs with *x* = 0.150, *M*_*H*_ increased which opposes the decrease in their *M*_*s*_ and this is due to the larger particle size with larger blocking temperature. The values of (*α*) and (*δ*) are constants with average value equals 0.3 and 0.6, respectively. This is predicted since the second part of Eq. 4 is related to the change with the magnetic field which is now constant (200 Oe). The values of *T*_*C*_ for different samples are approximately the same as recorded experimentally. *γ* is a constant with a value equals 1 which is the same as in Bloch law. *β* is also almost a constant since it is related to the material with an average value of 8.

## Conclusions

Sn_*x*_Fe_3-2/3*x*_O_4_ nanoparticles (12–50 nm) with *x* = 0.000 to 0.0150 were prepared using co-precipitation method. The magnetization was measured using VSM while repeatedly heating and cooling the nanoparticles up to 900 K. An irreversible dip in magnetization with certain amplitude was noticed between two peaks at *T*_1_ and *T*_2_ during the first heating–cooling cycle. We relate the first peak to a chemical reduction of the oxidized layer at the surface of each nanoparticle. The second peak is referred to a crystal growth due to the sintering process. Coating the surface with Au prevent sintering process and the magnetic exchange interactions between nanoparticles. More stable magnetic behavior was obtained for the high concentration of dopant Sn^2+^ (*x* = 0.150) which make it more appropriate for high-temperature applications. Best fitting for *M*-*T* graphs were made using a phenomenological expression where a core-shell model with magnetization of a ferrimagnetic core obeying the modified Bloch law and a paramagnetic shell obeying Curie–Weiss law. The results presented in this work present a method to tune the magnetization characteristics of Fe_3_O_4_ nanoparticles by Sn^2+^ doping.

## Supplementary information

**Additional file 1: Figure S1.** TEM images of the Sn_*x*_Fe_3-2*x*/3_O_4_ samples with *x* = a: 0.000, b: 0.045, c: 0.090, d: 0.150 (The scale length = 20nm, insight: size distribution histogram of prepared nanoparticles. **Figure S2.** SEM/EDS X-ray elemental mapping of (a) Fe (green) (b) O (red) and (c) green/red overlay for pure Fe_3_O_4_ nanoparticles. **Figure S3.** SEM/EDS X-ray elemental mapping of (a) Fe (green) (b) O (red) (c) Sn (yellow) and (d) green/red/yellow overlay.

## Data Availability

Supplementary information file

## Abbreviations

NPs: Nanoparticles
VSM: Vibrating sample magnetometer
PPMS: Physical property measurement system
HRTEM: High-resolution transmission electron microscope
XPS: X-ray photoemission spectroscopy
FTIR: Fourier transform infrared
XRD: X-ray diffraction
FC: Field cooling
ZFC: Zero field cooling
