# Supplementary material for: Sn2+ Doping: A Strategy for Tuning of Fe3O4 Nanoparticles Magnetization Dipping Temperature/Amplitude, Irreversibility, and Curie Point
Source: Nanoscale Res Lett. 2020 Oct 1;15:192. doi: 10.1186/s11671-020-03423-9 (PMC7530164; doi:10.1186/s11671-020-03423-9)
Supplement: Supplementary file 1 — Additional file 1: Figure S1. TEM images of the SnxFe3-2x/3O4 samples with x = a: 0.000, b: 0.045, c: 0.090, d: 0.150 (The scale length = 20nm, insight: size distribution histogram of prepared nanoparticles. Figure S2. SEM/EDS X-ray elemental mapping of (a) Fe (green) (b) O (red) and (c) green/red overlay for pure Fe3O4 nanoparticles. Figure S3. SEM/EDS X-ray elemental mapping of (a) Fe (green) (b) O (red) (c) Sn (yellow) and (d) green/red/yellow overlay. [file 11671_2020_3423_MOESM1_ESM.docx]

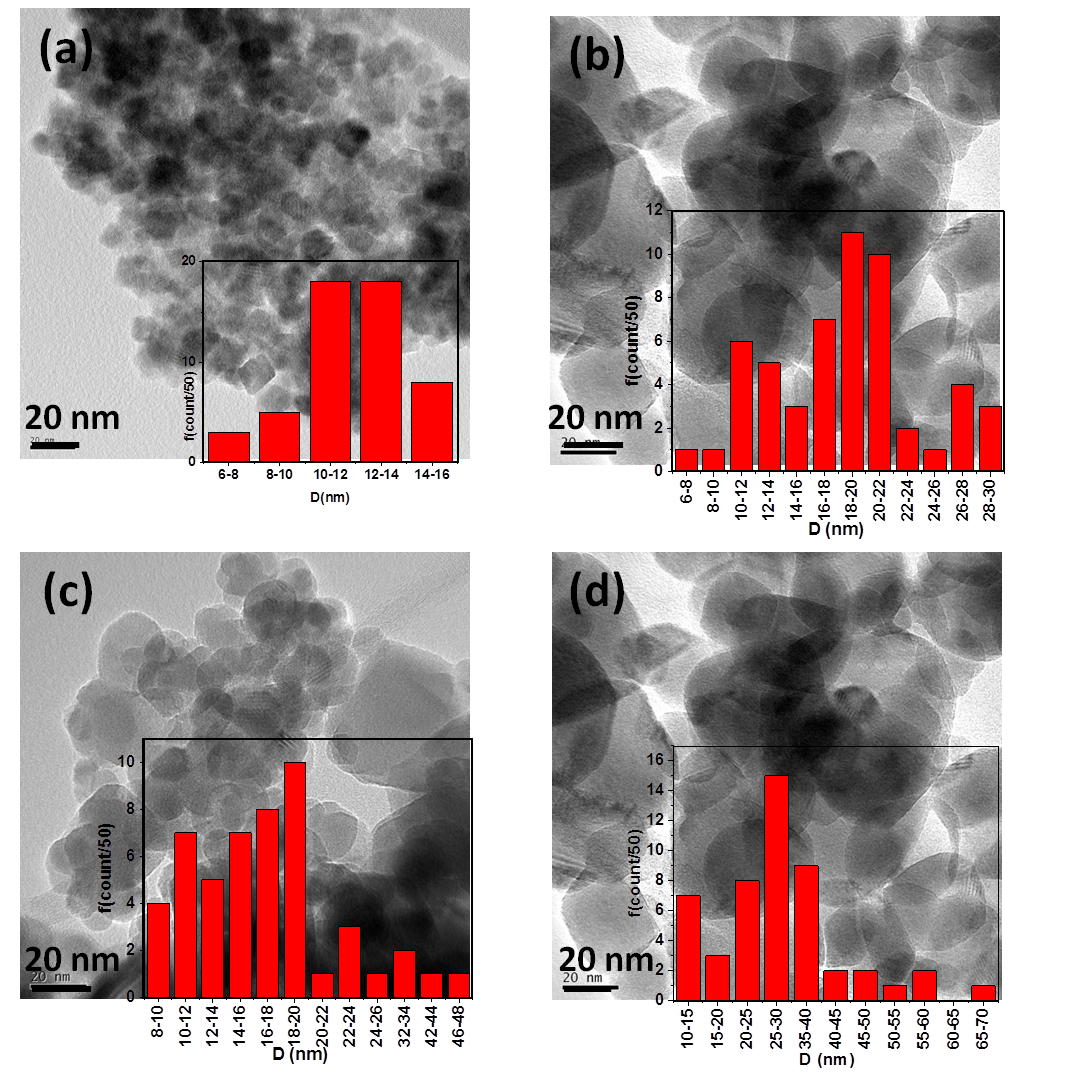


**Fig. S1** TEM images of the Sn*_x_*Fe_3-2_*_x_*_/3_O_4_ samples with *x* = a: 0.000, b: 0.045, c: 0.090, d: 0.150 (The scale length = 20nm, insight: size distribution histogram of prepared nanoparticles.


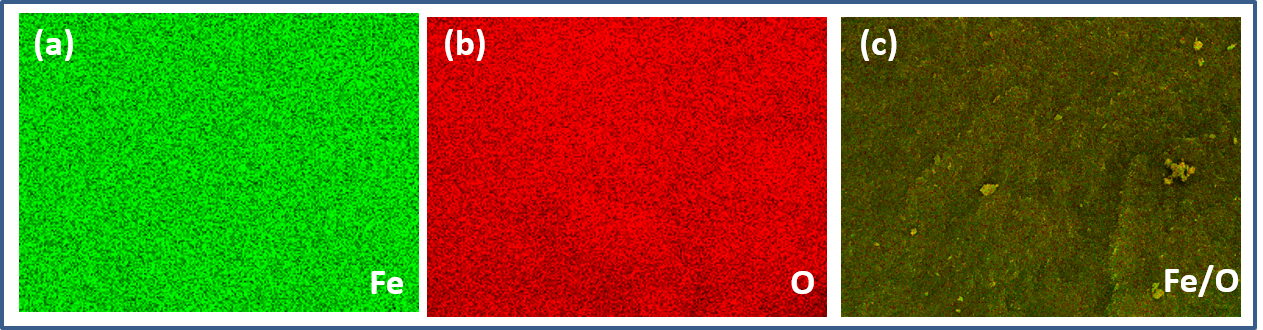


**Fig. S2** SEM/EDS X-ray elemental mapping of (a) Fe (green) (b) O (red) and (c) green/red overlay for pure Fe_3_O_4_ nanoparticles.


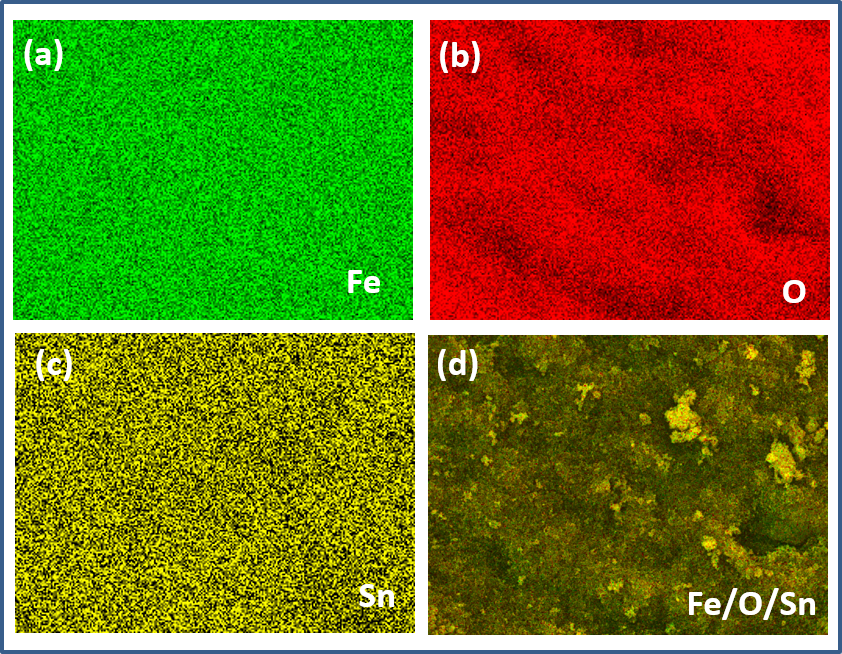


**Fig. S3** SEM/EDS X-ray elemental mapping of (a) Fe (green) (b) O (red) (c) Sn (yellow) and (d) green/red/yellow overlay.
